# Interferon regulatory factor-1 together with reactive oxygen species promotes the acceleration of cell cycle progression by up-regulating the cyclin E and CDK2 genes during high glucose-induced proliferation of vascular smooth muscle cells

**DOI:** 10.1186/1475-2840-12-147

**Published:** 2013-10-14

**Authors:** Xi Zhang, Long Liu, Chao Chen, Ya-Li Chi, Xiang-Qun Yang, Yan Xu, Xiao-Tong Li, Shi-Lei Guo, Shao-Hu Xiong, Man-Ru Shen, Yu Sun, Chuan-Sen Zhang, Kai-Meng Hu

**Affiliations:** 1 Institute of Biomedical Engineering, Second Military Medical University, Shanghai, China; 2 Changhai Hospital of Traditional Chinese Medicine, Second Military Medical University, Shanghai, China; 3 Department of Radiology, General Hospital of Beijing Military Region, Beijing, China; 4 Eastern Hepatobiliary Surgery Hospital, Second Military Medical University, Shanghai, China; 5 Nanjing RegeneCure Biotech, Nanjing, China; 6 Qingpu Branch of Zhongshan Hospital, Fudan University, Shanghai, China; 7 Histology and Embryology Department, Second Military Medical University, Shanghai, China

**Keywords:** Vascular smooth muscle cells, Cell proliferation, Interferon regulatory factor 1, Reactive oxygen species, Cell cycle

## Abstract

**Background:**

The high glucose-induced proliferation of vascular smooth muscle cells (VSMCs) plays an important role in the development of diabetic vascular diseases. In a previous study, we confirmed that Interferon regulatory factor-1 (Irf-1) is a positive regulator of the high glucose-induced proliferation of VSMCs. However, the mechanisms remain to be determined.

**Methods:**

The levels of cyclin/CDK expression in two cell models involving Irf-1 knockdown and overexpression were quantified to explore the relationship between Irf-1 and its downstream effectors under normal or high glucose conditions. Subsequently, cells were treated with high glucose/NAC, normal glucose/H_2_O_2_, high glucose/U0126 or normal glucose/H_2_O_2_/U0126 during an incubation period. Then proliferation, cyclin/CDK expression and cell cycle distribution assays were performed to determine whether ROS/Erk1/2 signaling pathway was involved in the Irf-1-induced regulation of VSMC growth under high glucose conditions.

**Results:**

We found that Irf-1 overexpression led to down-regulation of cyclin D1/CDK4 and inhibited cell cycle progression in VSMCs under normal glucose conditions. In high glucose conditions, Irf-1 overexpression led to an up-regulation of cyclin E/CDK2 and an acceleration of cell cycle progression, whereas silencing of Irf-1 suppressed the expression of both proteins and inhibited the cell cycle during the high glucose-induced proliferation of VSMCs. Treatment of VSMCs with antioxidants prevented the Irf-1 overexpression-induced proliferation of VSMCs, the up-regulation of cyclin E/CDK2 and the acceleration of cell cycle progression in high glucose conditions. In contrast, under normal glucose conditions, H_2_O_2_ stimulation and Irf-1 overexpression induced cell proliferation, up-regulated cyclin E/CDK2 expression and promoted cell cycle acceleration. In addition, overexpression of Irf-1 promoted the activation of Erk1/2 and when VSMCs overexpressing Irf-1 were treated with U0126, the specific Erk1/2 inhibitor abolished the proliferation of VSMCs, the up-regulation of cyclin E/CDK2 and the acceleration of cell cycle progression under high glucose or normal glucose/H_2_O_2_ conditions.

**Conclusions:**

These results demonstrate that the downstream effectors of Irf-1 are cyclin E/CDK2 during the high glucose-induced proliferation of VSMCs, whereas they are cyclin D1/CDK4 in normal glucose conditions. The Irf-1 overexpression-induced proliferation of VSMCs, the up-regulation of cyclin E/CDK2 and the acceleration of cell cycle progression are associated with ROS/Erk1/2 signaling pathway under high glucose conditions.

## Introduction

The high glucose-induced proliferation of vascular smooth muscle cells (VSMCs) plays an important role in the development of diabetic vascular diseases. However, the molecular mediators responsible for VSMC proliferation remain to be determined. We have previously shown that overexpression of Interferon regulatory factor-1 (Irf-1) accelerates the proliferation of VSMCs and that down-regulation of Irf-1 expression significantly depresses the proliferation of VSMCs under high glucose conditions. Irf-1 has also been shown to be a positive regulator of the high glucose-induced proliferation of VSMCs. Interestingly, our previous data demonstrated that Irf-1 overexpression has an anti-proliferative effect under normal glucose conditions, and it was suggested that the contradictory results were caused by high glucose levels [1]. Irf-1, a transcriptional regulator, most likely causes this discrepancy by regulating downstream effector genes under high glucose conditions that are different from the genes it regulates under normal physiological conditions.

Originally, Irf-1 was known as a transcription factor that recognizes regulatory elements in the promoters of interferon-beta and some interferon-inducible genes. Now by increasing evidence, the transcription factor has been defined as having the effect of regulating proliferation of various cell types including tumor cells and somatic cells [2-4]. Several potential downstream mediators of the growth-regulatory activity of Irf-1 have been identified, which include p53, p21, cyclins and cyclin-dependent kinase (CDK) [5-8]. Cyclins and CDK are downstream effector genes that control cell cycle checkpoints, suggesting that Irf-1 is involved in cell cycle regulation. The involvement of Irf-1 in cell cycle regulation may partly explain the effect of this gene in regulating VSMC growth.

Cell-cycle progression is regulated by cyclins and CDK. In early G1, certain events can promote transcription of cyclin D protein, which forms a cyclin D/CDK4 complex that phosphorylates the retinoblastoma (Rb), resulting in the gene expression and the formation of cyclin E/CDK2 complex. The cyclin E-CDK2 phosphorylates a broad variety of proteins and promotes cell-cycle progression to late G1, leading to the formation of the cyclin A/CDK2 complex, which promotes cell-cycle progression through the G1/S phase into S phase. In the above-mentioned signaling cascade, cyclin D/CDK4 and cyclin E/CDK2 are known as two key points that promote the G1/S-phase transition in cell cycle regulation [9]. However, the relationship between Irf-1 and the cyclins/CDK during the high glucose-induced proliferation of VSMCs needs to be confirmed. Furthermore, the glucose-dependent mechanism by which Irf-1 acts as a positive or negative regulator of VSMC growth needs to be elucidated.

In this study, two cell models involving Irf-1 knockdown and overexpression were established as previously described [1]. The levels of cyclin/CDK expression in two cell models were quantified to explore the relationship between Irf-1 and its downstream effectors associated with cell cycle regulation under normal or high glucose conditions. Subsequently, cells were treated with high glucose/N-acetyl-cysteine (NAC) and normal glucose/H_2_O_2_ or high glucose/U0126 and normal glucose/H_2_O_2_/U0126 during an incubation period. And then proliferation, cyclin/CDK expression and cell cycle distribution assays were performed to determine whether reactive oxygen species (ROS)/Erk1/2 signaling pathway was involved in the Irf-1-induced regulation of VSMC growth under high glucose conditions. In addition, the levels of phospho-Erk1/2 expression in two cell models involving Irf-1 knockdown and overexpression were quantified to explore the relationship between Irf-1 and activation of Erk1/2 under high glucose or ROS stimulation.

## Methods

### Cell culture and gene transfer

All experimental procedures were conducted in conformity with the institutional guidelines for the care and use of laboratory animals of the Second Military Medical University (Shanghai, China) and conformed to the National Institutes of Health Guide for the Care and Use of Laboratory Animals. The methods employed for VSMC culture and gene transfer were described previously [1]. Briefly, VSMCs were grown from explants of the thoracic aorta from SD rats. Cells between passages 3 and 6 were used in the experiments. Complementary oligonucleotide sequences were designed as small RNA interfering sequences according to IRF-1 cDNA sequences. The sequences were analyzed via BLAST searches to ensure that they did not show significant sequence homology to other genes. Small RNA interfering sequences (siIRF-1) were: sense, 5′ -GCC CAA CUC UCU ACU GUC Utt-3′; antisense, 5′ -AGA CAG UAG AGA GUU GGG Ctt-3′. The lentiviral vector pGCsi-FU-Irf-1 was obtained by inserting the siIRF-1 into pGC-FU-GFP (GeneChem, Shanghai, China), and the lentiviral vector pGC-FU-Irf-1 was obtained by subcloning the full-length Irf-1 cDNA (GenBank accession No. NM_012591.1) into pGC-FU-GFP (Additional file 1). The Irf-1 sequence was purchased commercially from GeneChem.

The pGCsi-FU-Irf-1 or pGC-FU-Irf-1 plasmid together with pHelper 1.0 and pHelper 2.0 plasmids (GeneChem) were cotransfected into 293 T cells using Lipofectamine 2000 (Invitrogen, Carlsbad, CA) to produce lentiviral stocks. The blank vector pGC-FU-GFP was utilized as a negative control. After the viral titers were determined, lentiviral particles were used to infect VSMCs.

### Intracellular ROS measurement

ROS levels were determined by measuring the oxidative conversion of DCFH-DA to fluorescent compound dichlorofluorescin. VSMCs in 96-well plates were incubated with normal glucose (5.5 mM) or high glucose (25 mM) solution for 2, 4 and 6 days. Thereafter, the medium was removed and replaced with serum-free medium. DCFH-DA (Beyotime Institute of Biotechnology, Jiangsu, China) stock solution (10 mM) was diluted 1000-fold in serum-free medium, and was added to each well of 96-well plates (10 uM). The cells were incubated for 25 min and then DCF fluorescence was determined at 520 nm following excitation with 488 nm light from an argon laser with a FACscan. The results were expressed as relative fluorescence intensity per 10^4^ cells.

### Cell proliferation and cycle assay

Transfected VSMCs were seeded onto 6-well plates at a density of 1 × 10^5^ cells/well in 2 ml of DMEM containing 10% FBS with normal glucose (5.5 mM). After 24 h, G0/early G1 synchronization was achieved through serum deprivation (0% FBS). Subsequently, the medium was switched to DMEM containing 10% FBS with normal glucose/H_2_O_2_ (0.06 mmol/L), normal glucose/H_2_O_2_/U0126 (10 uM), high glucose (25 mM)/NAC (20 mmol/L) and high glucose/U0126, respectively. For the measurement of cell proliferation under normal glucose and high glucose conditions, after a 5-day incubation period, cell growth and viability were analyzed via cell counting and using the EdU assay. Additionally, flow cytometry was employed to detect the cell cycle distribution of transfected VSMCs in normal glucose/H_2_O_2_, normal glucose/H_2_O_2_/U0126, high glucose/NAC and high glucose/U0126 conditions, respectively. The obtained data are expressed as the mean ± SD. Differences were assessed using Dunnett’s test, and a value of P < 0.05 was considered statistically significant.

### Western blot analysis of cyclins and CDK

Transfected VSMCs, including cells transfected with pGCsi-FU-Irf-1, pGCFU-Irf-1 or the pGC-FU vector, were incubated under normal glucose (5.5 mM), normal glucose/H_2_O_2_ (0.06 mmol/L), normal glucose/H_2_O_2_/U0126 (10 uM), high glucose (25 mM), high glucose/NAC (20 mmol/L) and high glucose/U0126 conditions, respectively. After 5 days, the cells were collected and lysed in RIPA lysis buffer (Beyotime). Next, the extracted proteins were resolved via SDS–PAGE and electroblotted onto PVDF membranes (Beyotime). The proteins of interest were detected with anti-cyclin D1, anti-cyclin E, anti-CDK2 or anti-CDK4 using the ECL (Beyotime) chemiluminescence detection system. Band intensities were analyzed using Multi-Analyst software. β-actin levels were used to normalize the signal intensity. The intensity data were then subjected to statistical analysis, and the differences were assessed via Dunnett’s test. A value of P < 0.05 was considered statistically significant.

### Immunofluorescence staining

Transfected VSMCs, including cells transfected with pGCsi-FU-Irf-1, pGCFU-Irf-1 or the pGC-FU vector, were incubated under normal glucose (5.5 mM), normal glucose/H_2_O_2_ (0.06 mmol/L), high glucose (25 mM), high glucose/NAC (20 mmol/L) conditions, respectively. After 5 days, the cells were plated at 50% confluence in 12-well plates, rinsed twice with PBS, and fixed in 95% ethanol for 30 min at room temperature. Rabbit anti phospho-Erk1/2 (Cell Signaling Technology, Inc.) was applied to incubate the cells at 4°C overnight. After incubation of the cells with secondary antibodies of anti- rabbit IgG-TRITC (Sigma–Aldrich, Inc.) used at a 1:100 dilution for 60 min at room temperature, fluorescence imaging was visualized with an Olympus IX70 microscope. The percentage of positive cells was determined by counting positive staining cells and total cells in one visual field under a microscope. At least three fields (around 300 cells) were counted in each sample. Average data are presented as means ± SD and compared with Dunnett-test.

## Results

### Irf-1 overexpression decreases the proliferation activity, inhibits cell cycle progression, and down-regulates cyclin D1/CDK4 expression in VSMCs under normal glucose conditions

We have reported previously that Irf-1 overexpression significantly depresses the proliferation of VSMCs under normal glucose conditions [1]. The finding is consistent with the results obtained in this study, which showed that under normal glucose conditions, more than 88% of VSMCs transfected with pGC-FU-Irf-1 were in the G0/G1 phase of the cell cycle, and the percentage of cells in S and G2/M phases was significantly lower compared to untransfected cells (Table 1). These results indicate that Irf-1 overexpression decreased the proliferation activity and inhibited cell cycle progression in VSMCs.

**Table 1:** Analysis of VSMCs cycle distribution by flow cytometry

		**Untransfected VSMCs (%)**	**VSMCs transfected with pGC-FU (%)**	**VSMCs transfected with pGCsi-FU-Irf-1 (%)**	**VSMCs transfected with pGC-FU-Irf-1 (%)**
High glucose	G0/G1	64.8 ± 2.7	65.0 ± 2.9	82.5 ± 3.2*	51.0 ± 2.4*
	S	21.2 ± 1.2	20.9 ± 1.4	10.5 ± 1.1*	29.8 ± 1.6*
	G2/M	13.9 ± 1.3	14.1 ± 1.7	6.9 ± 0.9*	19.3 ± 1.7*
High glucose/NAC	G0/G1	69.5 ± 2.7	69.9 ± 3.0	69.4 ± 1.3^#^	71.1 ± 3.4^#^
	S	18.7 ± 1.6	19.1 ± 1.2	18.3 ± 1.5^#^	17.9 ± 2.1^#^
	G2/M	11.7 ± 1.5	11.1 ± 1.4	12.3 ± 1.3^#^	10.9 ± 2.1^#^
Normal glucose	G0/G1	80.8 ± 3.3	80.3 ± 2.9	81.5 ± 3.7	88.4 ± 3.5*
	S	11.8 ± 1.2	12.2 ± 1.3	11.3 ± 1.0	7.3 ± 1.1*
	G2/M	7.3 ± 1.1	7.5 ± 1.0	7.3 ± 1.2	4.2 ± 0.7*
Normal glucose/H_2_O_2_	G0/G1	72.0 ± 2.9^	73.4 ± 3.1^	82.5 ± 3.2*	55.5 ± 2.3*^
	S	17.1 ± 2.2^	16.8 ± 1.7^	11.3 ± 1.2*	27.9 ± 2.3*^
	G2/M	10.8 ± 1.3^	9.9 ± 1.7^	6.1 ± 1.0*	16.7 ± 1.9*^
High glucose/U0126	G0/G1	71.3 ± 2.8^#^	72.5 ± 3.1^#^	70.6 ± 1.9^#^	69.8 ± 3.6^#^
	S	17.9 ± 1.6^#^	17.4 ± 2.0^#^	16.5 ± 1.6^#^	18.1 ± 1.9^#^
	G2/M	10.9 ± 1.7^#^	10.2 ± 1.5^#^	12.8 ± 2.8^#^	12.0 ± 2.6^#^
Normal glucose/H_2_O_2_/U0126	G0/G1	70.3 ± 2.1^	68.9 ± 3.6^	69.8 ± 2.3^	70.9 ± 3.2^
	S	17.5 ± 1.8^	18.5 ± 1.6^	18.4 ± 1.7^	17.8 ± 2.1^
	G2/M	12.1 ± 1.7^	12.5 ± 1.5^	11.9 ± 1.4^	11.4 ± 1.9^

Values are mean ± SD from triplicate determinations repeated in 4 separate experiments.

*P < 0.05 vs. corresponding values in untransfected VSMCs; ^P < 0.05 vs. corresponding values in VSMCs under normal glucose; ^#^P < 0.05 vs. corresponding values in VSMCs under high glucose.

**Figure 1:**
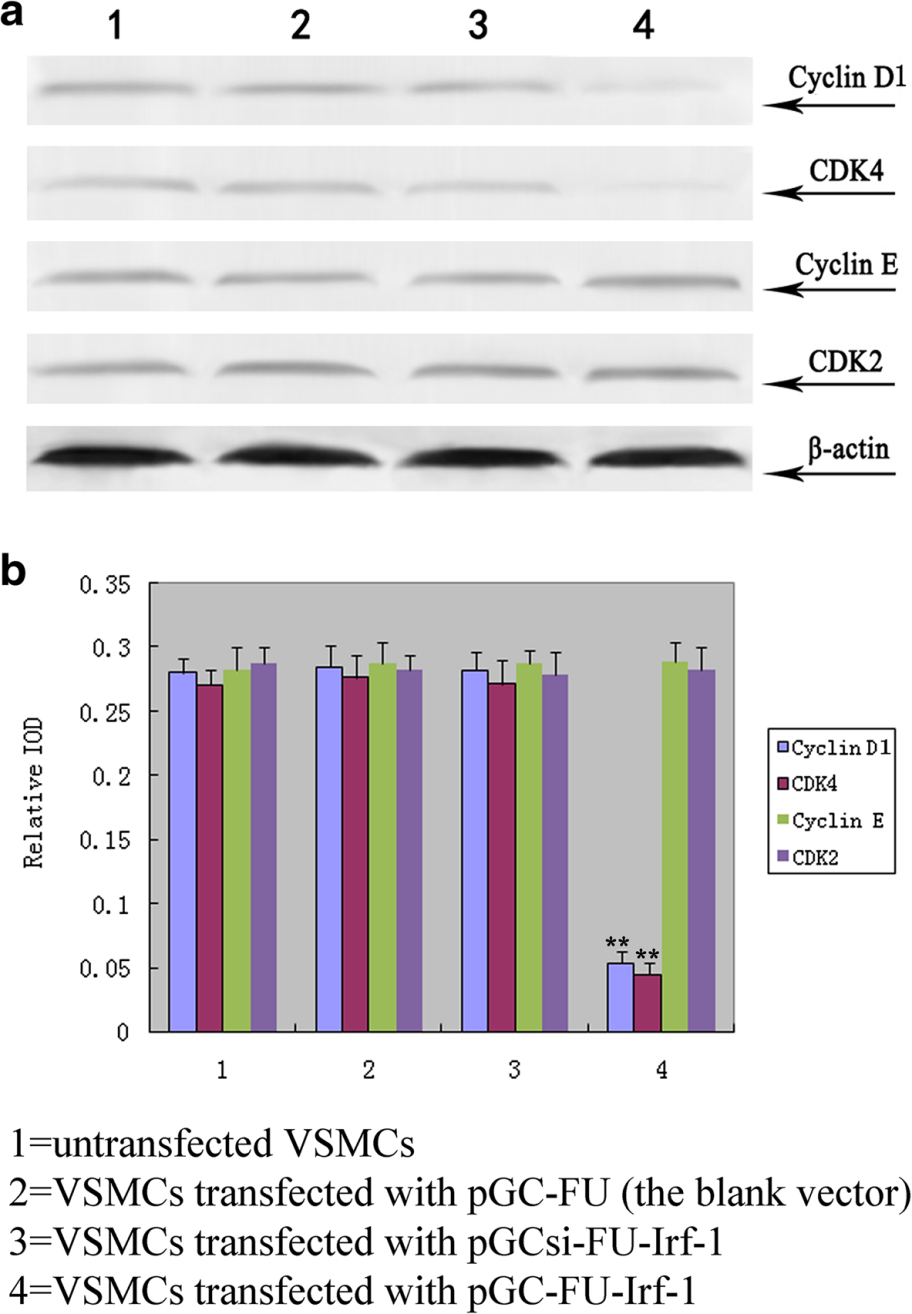
**Examination of the expression of cyclins/CDK in transfected VSMCs under normal glucose. a**: Western blot analysis of cyclin D1, CDK4, cyclin E and CDK2 after 5 days of incubation of the transfected VSMCs with normal glucose; **b**: quantitative assessment of cyclin/CDK protein levels through integrated optical density analyses. *P <0.05; **P <0.01 versus corresponding values in untransfected VSMCs.

To determine the effect of Irf-1 overexpression on cyclins and CDK under normal glucose conditions, VSMCs were transfected with pGCFU-Irf-1, and the expression of cyclin D1, cyclin E, CDK2 and CDK4 was examined via Western blot analysis. The results showed that the expression of cyclin D1 and CDK4 in VSMCs transfected with pGC-FU-Irf-1 was significantly lower than the levels in untransfected VSMCs or VSMCs transfected with the blank pGC vector (pGC-FU) (P < 0.01, n = 5; Figure 1), indicating that Irf-1 overexpression down-regulated cyclin D1 and CDK4 expression under normal glucose conditions. In contrast, no significant difference was observed in the expression of cyclin E or CDK2 between VSMCs transfected with pGC-FU-Irf-1 and control cells under normal glucose conditions (P > 0.05, n = 5; Figure 1).

### Manipulation of Irf-1 levels affects the proliferation activity, the cell cycle progression, the expression of cyclin E/CDK2 in VSMCs in the presence of high glucose

It has been shown in the previous study that overexpression of Irf-1 accelerates the proliferation of VSMCs and that down-regulation of Irf-1 expression significantly depresses the proliferation of VSMCs under high glucose conditions [1]. In this study, the results showed that under high glucose conditions, VSMCs transfected with pGC-FU-Irf-1 displayed a significantly greater percentage of cells in S and G2/M phases, while cells transfected with pGCsi-FU-Irf-1 exhibited a lower percentage of cells in these phases compared to untransfected cells (Table 1). This means that overexpression of Irf-1 promoted cell cycle progression and silencing of Irf-1 caused the opposite effect under high glucose conditions.

**Figure 2:**
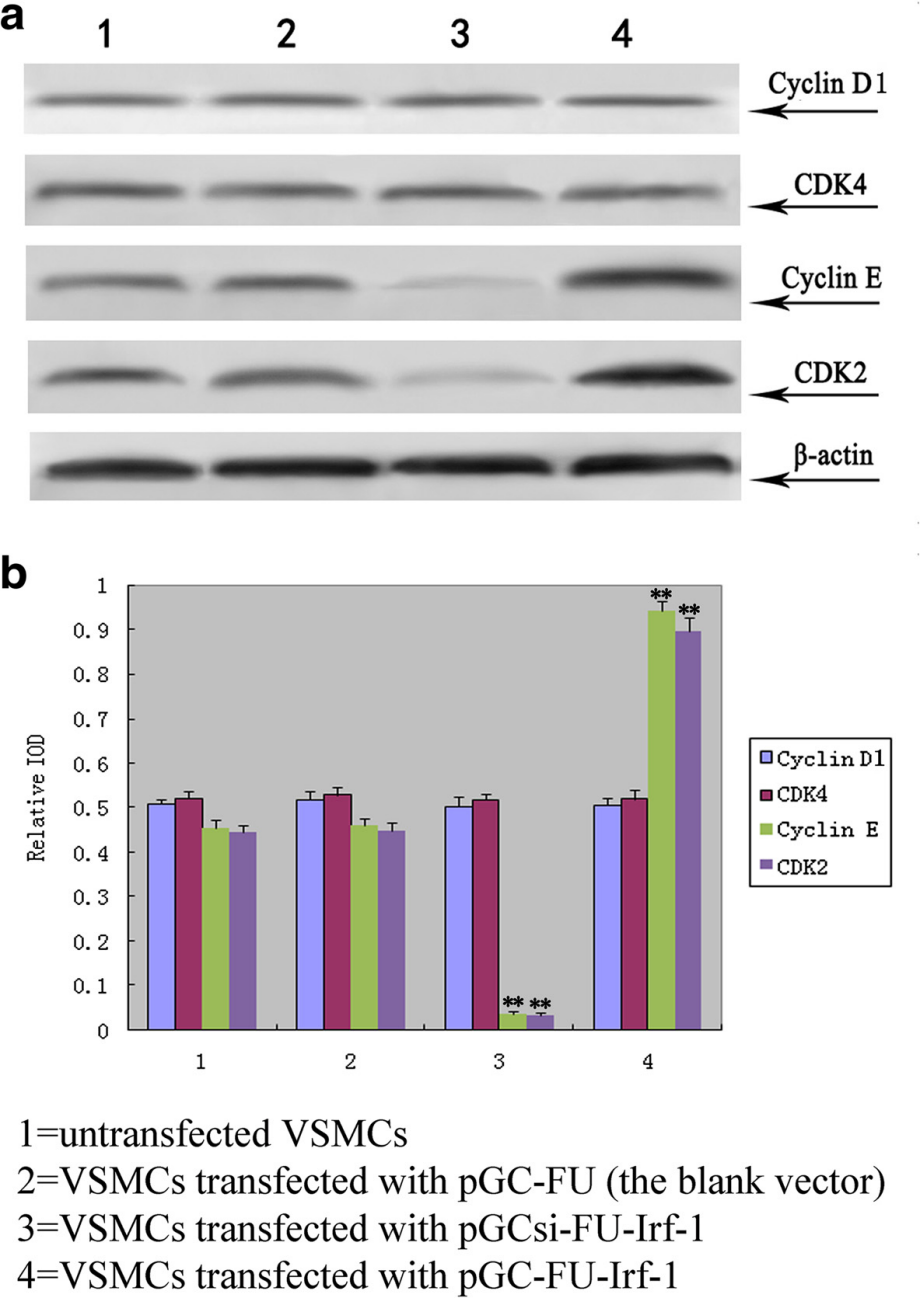
**Examination of the expression of cyclins/CDK in transfected VSMCs under high glucose. a**: Western blot analysis of cyclin D1, CDK4, cyclin E and CDK2 after 5 days of incubation of the transfected VSMCs with high glucose; **b**: quantitative assessment of cyclin/CDK protein levels through integrated optical density analyses. *P <0.05; **P <0.01 versus corresponding values in untransfected VSMCs.

To determine the effect of Irf-1 levels on cyclins and CDK under high glucose conditions, VSMCs were transfected with pGCsi-FU-Irf-1 and pGCFU-Irf-1, and the expression of cyclin D1, cyclin E, CDK2 and CDK4 was determined through Western blot assays. The results showed that the expression of cyclin E and CDK2 in VSMCs transfected with pGCsi-FU-Irf-1 was significantly lower than the expression recorded in untransfected VSMCs and VSMCs transfected with the blank pGC vector (P < 0.01, n = 5; Figure 2), indicating that the silencing of Irf-1 suppressed the expression of the two proteins under high glucose conditions. In contrast, the expression of cyclin E and CDK2 in VSMCs transfected with pGCFU-Irf-1 was significantly higher than the levels in untransfected VSMCs and VSMCs transfected with the blank pGC vector (P < 0.01, n = 5; Figure 2), indicating that Irf-1 overexpression up-regulated cyclin E and CDK2 under high glucose conditions. In addition, no significant difference was observed in the expression of cyclin D1 and CDK4 between VSMCs transfected with pGCsi-FU-Irf-1 or pGC-FU-Irf-1 and control cells under high glucose conditions (P > 0.05, n = 5; Figure 2).

### Relationship between ROS and Irf-1-mediated hyperglycemia-dependent VSMC proliferation

**Figure 3:**
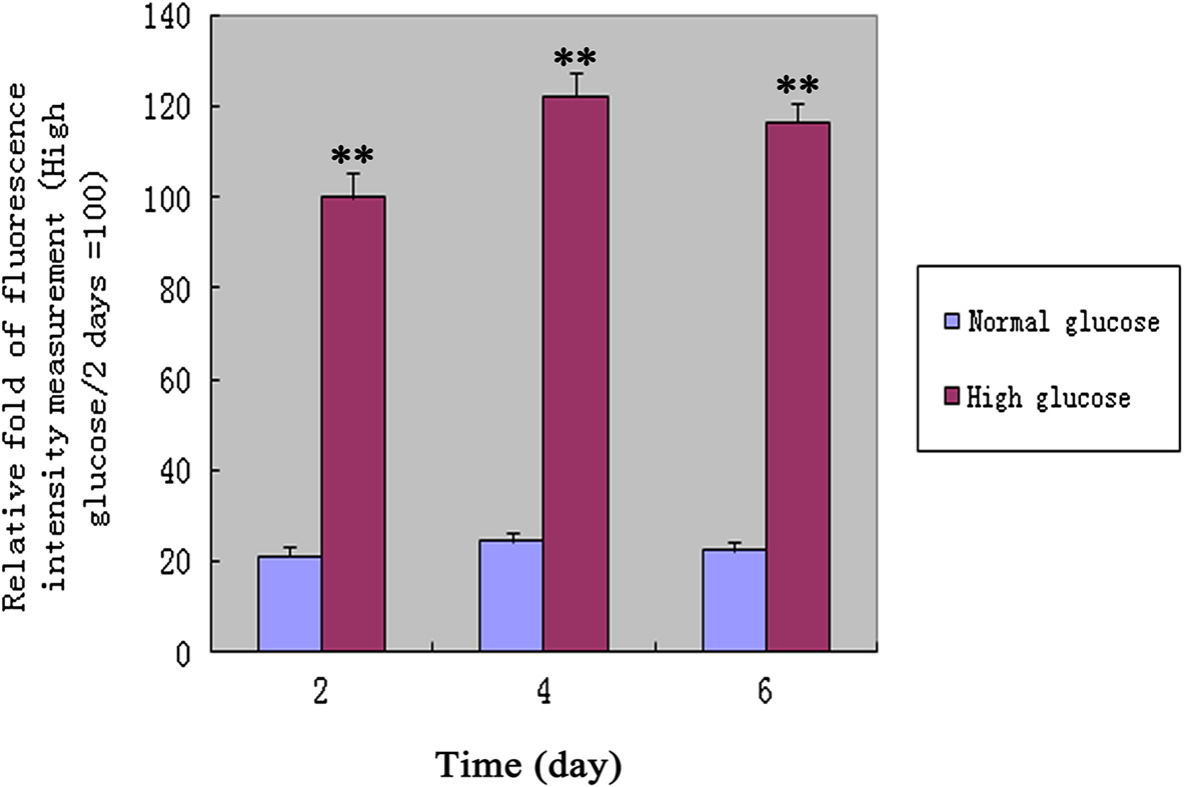
**The intracellular ROS production was measured by flow cytometry analysis under high glucose or normal glucose conditions.** Values are showed as relative fold of fluorescence intensity measurement (High glucose/2 days =100). *P <0.05; **P <0.01 versus corresponding values in VSMCs under normal glucose conditions. 2 days, 4 days and 6 days = VSMCs incubated with high glucose or normal glucose for 2 days, 4 days and 6 days.

ROS production in VSMCs was measured by flow cytometric analysis. The results showed that the level of intracellular ROS under high glucose conditions was higher than that under normal glucose conditions (P < 0.01, n = 12; Figure 3 and Table 2), which indicates that high glucose treatment increased significantly the level of intracellular ROS in VSMCs.

**Table 2:** Analysis of intracellular ROS levels by flow cytometry

	**2 days (Relative fold)**	**4 days (Relative fold)**	**6 days (Relative fold)**
High glucose	100.0 ± 5.7**	122.3 ± 5.1**	116.5 ± 4.2**
Normal glucose	21.2 ± 2.0	24.3 ± 2.3	22.4 ± 2.1

Values are mean ± SD from triplicate determinations repeated in 4 separate experiments. They are showed as relative fold of fluorescence intensity measurement (High glucose/2 days =100).

2 days, 4 days and 6 days = VSMCs incubated with high glucose or normal glucose for 2 days, 4 days and 6 days.

*P <0.05; **P <0.01 versus corresponding values in VSMCs under normal glucose conditions.

**Figure 4:**
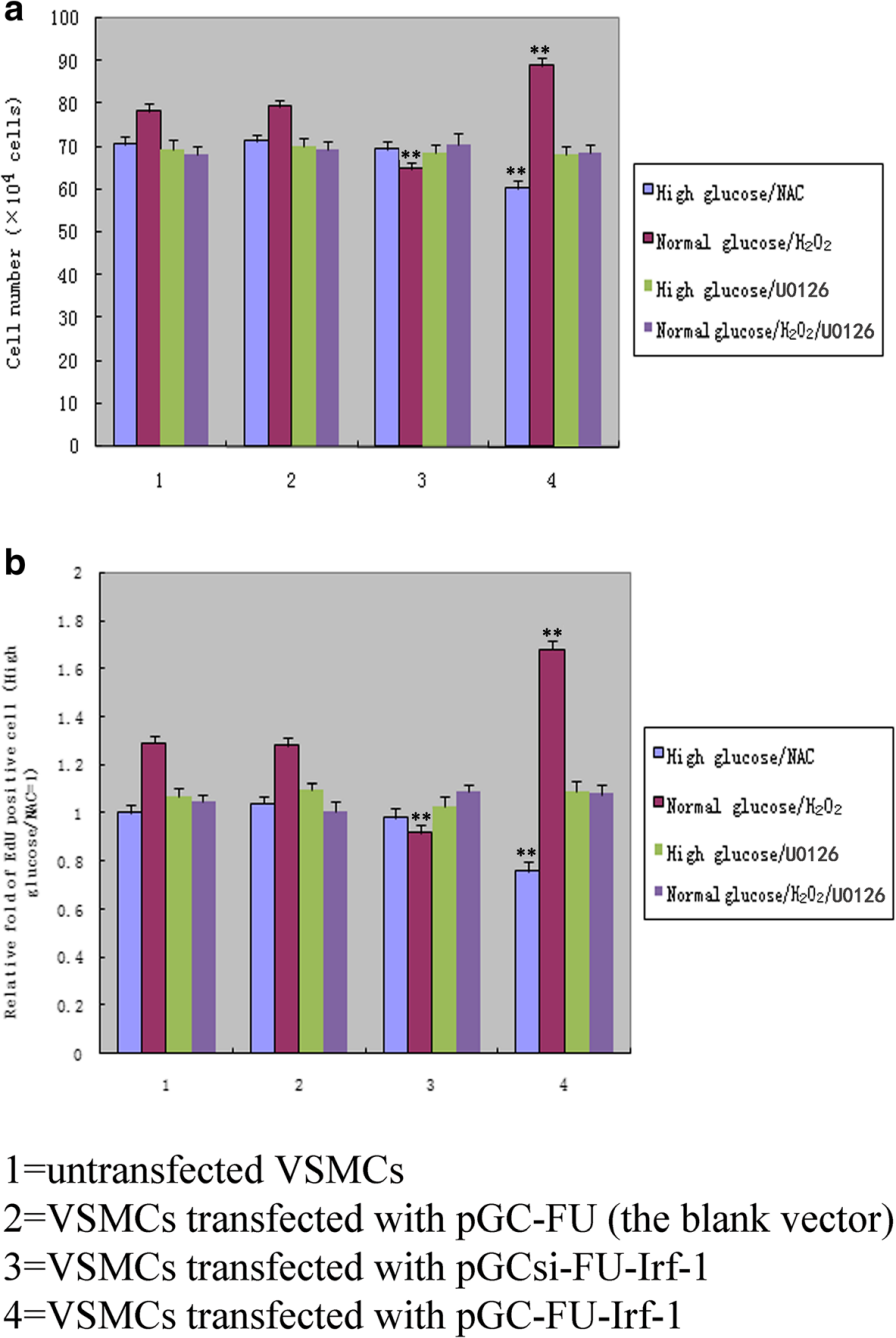
**Comparison of cell proliferation in transfected VSMCs following treatment with NAC, H**_**2**_**O**_**2 **_**or U0126. a**: cell numbers were counted with a hemocytometer; **b**: cell proliferation was also measured using the EdU assay; the presented data are the mean ± SD of triplicate determinations repeated in four separate experiments. High glucose/NAC = cells incubated with high glucose and NAC for 5 days; normal glucose/H_2_O_2_ = cells incubated with normal glucose and H_2_O_2_ for 5 days; High glucose/U0126 = cells incubated with high glucose and U0126 for 5 days; normal glucose/H_2_O_2_/U0126 = cells incubated with normal glucose, H_2_O_2_ and U0126 for 5 days. *P <0.05; **P <0.01 versus corresponding values in untransfected VSMCs.

When VSMCs were treated with NAC, no significant difference in cell numbers or proliferation activity was observed between VSMCs transfected with pGC-FU-Irf-1 and control cells under high glucose conditions (P > 0.05, n = 12; Figure 4). In contrast, following the addition of H_2_O_2_ to the cells, the number of pGC-FU-Irf-1-transfected VSMCs was significantly greater compared to untransfected VSMCs and VSMCs transfected with the blank pGC vector under normal glucose conditions (P < 0.01, n = 12; Figure 4). These findings are consistent with the results obtained using the EdU assay, which indicates that the proliferation activity of VSMCs transfected with pGC-FU-Irf-1 was increased significantly compared to untransfected VSMCs and VSMCs transfected with the blank pGC vector in the presence of H_2_O_2_ or ROS (P < 0.01, n = 12; Figure 4).

### ROS affects Irf-1-dependent cyclin E/CDK2 expression and cell cycle regulation under high glucose conditions

**Figure 5:**
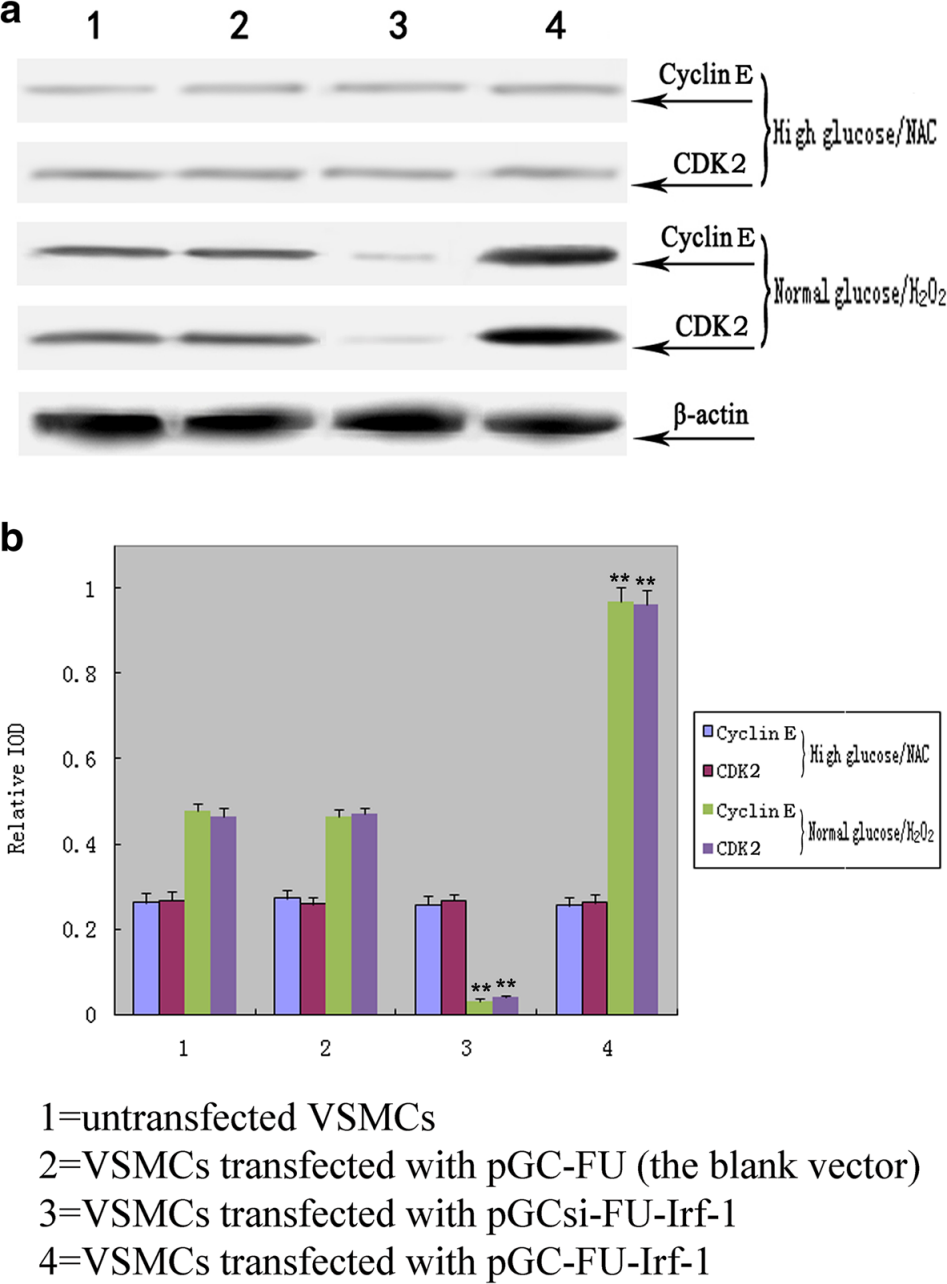
**Western blot analysis of cyclin E and CDK2 in transfected VSMCs following treatment with NAC or H**_**2**_**O**_**2**_**. a**: Western blot analysis of the expression of cyclin E and CDK2 protein levels in transfected VSMCs; **b**: quantitative assessment of cyclin E and CDK2 protein levels through integrated optical density analyses. High glucose/NAC = cells incubated with high glucose and NAC for 5 days; normal glucose/H_2_O_2_ = cells incubated with normal glucose and H_2_O_2_ for 5 days. *P <0.05; **P <0.01 versus corresponding values in untransfected VSMCs.

When VSMCs were treated with NAC, a potent antioxidant, no significant difference in the expression of cyclin E or CDK2 was observed between VSMCs transfected with pGC-FU-Irf-1 and control cells under high glucose conditions, indicating that NAC blocked the Irf-1-induced up-regulation of cyclin E and CDK2 under high glucose conditions (P > 0.05, n = 5; Figire 5). After the addition of H_2_O_2_ to the cells, the expression of the two proteins in VSMCs transfected with pGC-FU-Irf-1 was significantly higher than the expression levels observed in untransfected VSMCs and VSMCs transfected with the blank pGC vector under normal glucose conditions (P < 0.01, n = 5; Figure 5), indicating that the Irf-1-mediated up-regulation of cyclin E and CDK2 under high glucose conditions was regulated by ROS.

To determine the effect of Irf-1 levels and ROS on cell cycle regulation, the cell cycle distribution of VSMCs was estimated via flow cytometry. Under high glucose conditions, VSMCs transfected with pGC-FU-Irf-1 displayed a significantly greater percentage of cells in S and G2/M phases, while cells transfected with pGCsi-FU-Irf-1 exhibited a lower percentage of cells in these phases compared to untransfected cells. In contrast, transfected VSMCs treated with NAC/high glucose showed no significant difference in the percentage of S and G2/M phases compared to the control cells (Table 1). Under normal glucose conditions, more than 88% of VSMCs transfected with pGC-FU-Irf-1 were in the G0/G1 phase of the cell cycle, and the percentage of cells in S and G2/M phases was significantly lower compared to untransfected cells. In contrast, VSMCs transfected with pGC-FU-Irf-1 and treated with H_2_O_2_/normal glucose showed a significantly greater percentage of cells in S and G2/M phases, while a lower percentage of cells transfected with pGCsi-FU-Irf-1 were in these phases compared to untransfected cells (Table 1). These results indicate that the Irf-1-mediated cell cycle acceleration or arrest under high glucose conditions was regulated by ROS.

### Blocking Erk1/2 pathway affects Irf-1-dependent VSMC proliferation, cyclin E/CDK2 expression, and cell cycle regulation under high glucose conditions

**Figure 6:**
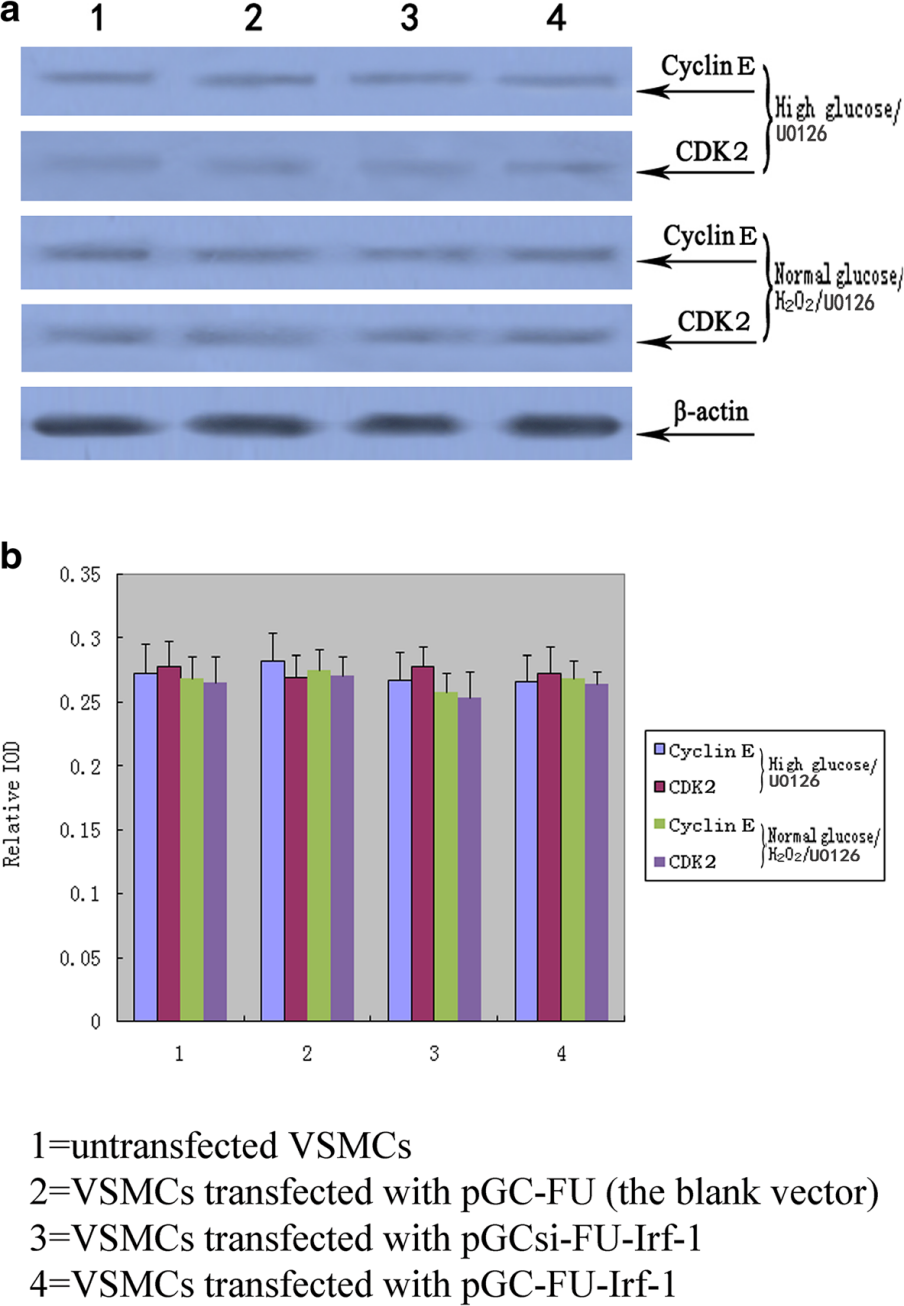
**Western blot analysis of cyclin E and CDK2 in transfected VSMCs following treatment with U0126. a**: Western blot analysis of the expression of cyclin E and CDK2 protein levels in transfected VSMCs; **b**: quantitative assessment of cyclin E and CDK2 protein levels through integrated optical density analyses. High glucose/U0126 = cells incubated with high glucose and U0126 for 5 days; normal glucose/H_2_O_2_/U0126 = cells incubated with normal glucose, H_2_O_2_ and U0126 for 5 days. *P <0.05; **P <0.01 versus corresponding values in untransfected VSMCs.

When VSMCs were treated with U0126, the specific Erk1/2 upstream kinase MEK inhibitor, no significant difference in cell proliferation activity, cyclin E/CDK2 expression, and cell cycle distribution was observed between VSMCs transfected with pGC-FU-Irf-1 and control cells under high glucose conditions. Similarly, there was no significant difference in the proliferation activity, cyclin E/CDK2 expression, and the cycle distribution between pGC-FU-Irf-1-transfected VSMCs and control cells with U0126 treatment under normal glucose/H_2_O_2_ conditions (P > 0.05, n = 12; Figure 4) (P > 0.05, n = 5; Figure 6) (Table 1). These findings indicate that blocking Erk1/2 pathway inhibited Irf-1-mediated ROS or hyperglycemia-dependent VSMC proliferation.

### Irf-1 regulates the activation of Erk1/2 in VSMC under high glucose or normal glucose/H_2_O_2_ conditions

**Figure 7:**
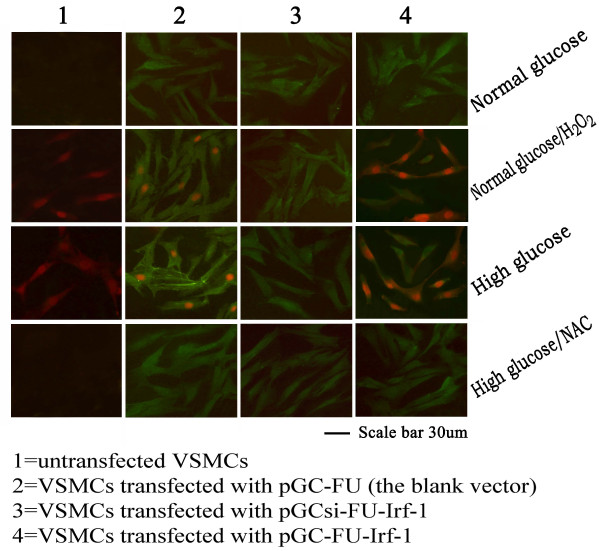
**Representative negative or positive staining of phospho-Erk1/2 in VSMCs.** High glucose = cells incubated with high glucose for 5 days; normal glucose = cells incubated with normal glucose for 5 days; High glucose/NAC = cells incubated with high glucose and NAC for 5 days; normal glucose/H_2_O_2_ = cells incubated with normal glucose and H_2_O_2_ for 5 days. Positive staining of phospho-Erk1/2 shows red and transfected VSMCs display green (GFP).

The phosphorylation of ERK1/2 (phospho-Erk1/2) was determined by immunofluorescence staining. The percentage of phospho-Erk1/2 positive cells was low about 2-3% under normal glucose or high glucose/NAC conditions, while the percentage was great about 26-27% under high glucose or normal glucose/H_2_O_2_ conditions. Furthermore, VSMCs transfected with pGC-FU-Irf-1 displayed a significantly greater percentage of phospho-Erk1/2 positive cells, while cells transfected with pGCsi-FU-Irf-1 exhibited a lower percentage of phospho-Erk1/2 positive cells compared to untransfected cells under high glucose or normal glucose/H_2_O_2_ conditions. In contrast, transfected VSMCs treated with high glucose/NAC or normal glucose showed no significant difference in the percentage of phospho-Erk1/2 positive cells compared to the control cells (Figure 7) (Table 3). These results indicate that overexpression of Irf-1 promoted the activation of Erk1/2 and silencing of Irf-1 caused the opposite effect under high glucose or normal glucose/H_2_O_2_ conditions.

**Table 3:** The percentage of phospho-Erk1/2 positive cells

		**Uuntransfected VSMCs (%)**	**VSMCs transfected with pGC-FU (%)**	**VSMCs transfected with pGCsi-FU-Irf-1 (%)**	**VSMCs transfected with pGC-FU-Irf-1 (%)**
Normal glucose	+	2.56 ± 0.33	2.92 ± 0.28	3.11 ± 0.34	2.65 ± .026
	++	0.32 ± 0.03	0.39 ± 0.04	0.45 ± 0.02	0.24 ± 0.06
Normal glucose + H_2_O_2_	+	19.7 ± 2.8^	20.5 ± 2.6^	2.89 ± 0.59*	33.7 ± 2.9*^
	++	6.1 ± 0.7^	6.3 ± 0.6^	0.43 ± 0.05*	34.7 ± 2.8*^
High glucose	+	21.8 ± 1.9	23.7 ± 2.2	3.27 ± 0.28*	34.5 ± 3.8*
	++	5.7 ± 0.8	6.2 ± 0.5	0.36 ± 0.03*	35.1 ± 3.2*
High glucose + NAC	+	3.04 ± 0.29^#^	3.17 ± 0.42^#^	3.13 ± 0.48	2.75 ± .034^#^
	++	0.51 ± 0.04^#^	0.31 ± 0.06^#^	0.48 ± 0.04	0.44 ± 0.05^#^

Values are mean ± SD from the determinations repeated in 4 separate experiments (12 samples). For one sample, the percentage of positive cells was determined by counting positive staining cells and total cells in three visual fields (around 3 × 300 cells).

*p < 0.05 vs. corresponding values in untransfected VSMCs; ^P < 0.05 vs. corresponding values in VSMCs under normal glucose; ^#^P < 0.05 vs. corresponding values in VSMCs under high glucose.

+ represents positive; ++ represents strong positive.

## Discussion

Hyperglycemia is a risk factor of cardiovascular morbidity and mortality in patients [10]. It has a very important significance for the clinic to clarify the hyperglycemia-dependent mechanisms responsible for cardiovascular diseases. In particular, molecular mediators responding for the High glucose-Induced diseases may prove to be a potential target gene for disease treatment [11,12]. At present, most of them remain to be determined. Irf-1, a molecular mediator for vascular diseases, was previously known as negative. However, several of recent studies have provided evidences that Irf-1 was not a negative mediator, but even a positive one [1,13,14].

Irf-1 displays tumor suppressor activity and is regarded as a negative regulator of cell growth through regulating downstream effector genes [3,15,16]. It is well known that cyclin D and CDK4 form a complex that promotes the G1/S-phase transition in cell cycle regulation. Kröger et al. [6] reported that Irf-1 inhibits cell growth by suppressing cyclin D/CDK4 gene transcription. Therefore, cyclin D/CDK4 may be the downstream effector genes involved in Irf-1-induced VSMC growth inhibition. In a previous study [1], we determined that Irf-1 overexpression in VSMCs has an anti-proliferative effect under normal glucose conditions, consistent with the tumor suppressor activity of Irf-1. In this study, we confirmed that Irf-1 overexpression leads to the down-regulation of cyclin D1/CDK4 and inhibits VSMC cell cycle progression under normal glucose conditions, suggesting that cyclin D1/CDK4 are downstream effector genes of Irf-1 with an antiproliferative effect on VSMCs.

In contrast to the antiproliferative effect of Irf-1/normal glucose on cells, we previously showed that Irf-1/high glucose has a pro-proliferative effect on VSMCs [1]. The likely mechanism underlying this discrepancy is that Irf-1, a transcriptional regulator, regulates downstream effector genes under high glucose conditions that are different from the genes it regulates under normal glucose conditions. Cyclin E and its catalytic partner, CDK2, are known to play important roles in the G1/S checkpoint of the cell cycle [17]. In this study, we found that Irf-1 overexpression up-regulated cyclin E/CDK2 expression and accelerated cell cycle progression. In contrast, silencing of Irf-1 suppressed the expression of these two proteins and inhibited the cell cycle during the high glucose-induced proliferation of VSMCs. This finding suggests that the downstream effector genes of Irf-1 are cyclin E/CDK2 and that they are positively regulated under high glucose conditions, while cyclin D1/CDK4 are negatively regulated under normal glucose conditions. In the cyclins/CDK signaling cascade, cyclin D/CDK4 and cyclin E/CDK2 are known as sequential activation that promotes the G1/S-phase transition. Consequently, through downstream effectors cyclin D/CDK4, Irf-1 could exert an anti-proliferative effect under normal glucose conditions, but through cyclin E/CDK2 it could accelerate proliferation under high glucose conditions.

Irf-1 is regarded as a negative regulator of cell growth under typical-state/normal glucose conditions. Why does Irf-1 up-regulate cyclin E/CDK2 and promote the proliferation of VSMCs under high glucose conditions? Several studies have attributed this difference to ROS, based on the notion that ROS influence cell proliferation and promote cell cycle progression by activating cyclins/CDK, as ROS levels increase steadily during the high glucose-induced proliferation of VSMCs [18-21]. Accordingly, the possibility has been raised that ROS are involved in the Irf-1/high glucose-induced proliferation of VSMCs. In the present study, we found that high glucose treatment increased significantly the level of intracellular ROS in VSMCs and when VSMCs overexpressing Irf-1 under high glucose conditions were treated with antioxidants, a proliferation of VSMCs, an up-regulation of cyclin E/CDK2 and an acceleration of cell cycle progression was not observed. In contrast, H_2_O_2_ stimulation under normal glucose conditions in Irf-1-overexpressing cells caused cell proliferation, an up-regulation of cyclin E/CDK2 and cell cycle acceleration, suggesting that under ROS or H_2_O_2_ stimulation, the positive regulatory activity of Irf-1 may play a role in cell growth.

Activation of Erk1/2 MAPK pathway is essential for VSMC growth. ROS has been shown, via Erk1/2 pathway, to mediate the cell proliferative effects [9,18]. In this study, we observed that when VSMCs overexpressing Irf-1 were treated with U0126, the specific Erk1/2 inhibitor abolished the proliferation of VSMCs, the up-regulation of cyclin E/CDK2 and the acceleration of cell cycle progression under high glucose or normal glucose/H_2_O_2_ conditions. These results indicate that Erk1/2 activation is required for Irf-1-mediated hyperglycemia-dependent VSMC proliferation. Taken together, above findings suggest the pathway of Irf-1-mediated hyperglycemia-dependent VSMC proliferation. First, high glucose stimulation increases the level of intracellular ROS in VSMCs. Subsequently, via Erk1/2 pathway, the increased ROS induces the up-regulation of cyclin E/CDK2 which leads to the proliferation of VSMCs and the acceleration of cell cycle. Because overexpression of Irf-1 promotes the activation of Erk1/2 under high glucose conditions, the positive regulatory activity of Irf-1 could play a role in hyperglycemia-dependent VSMC proliferation.

In conclusion, we demonstrated that the downstream effector genes of Irf-1 are cyclin E/CDK2 during the high glucose-induced proliferation of VSMCs, whereas they are cyclin D1/CDK4 under normal glucose conditions. Irf-1 overexpression-induced VSMC proliferation, an up-regulation of cyclin E/CDK2 and an acceleration of cell cycle progression are associated with ROS/ERK1/2 signaling pathway under high glucose conditions. The findings of the present study may prove significant in furthering our understanding of the development and regulatory mechanisms of diabetic vascular diseases.

## Abbreviations

Irf-1: Interferon regulatory factor-1; VSMCs: Vascular smooth muscle cells; siRNA: RNA interfering sequences; FBS: Fetal bovine serum; CDK: Cyclin-dependent kinase; NAC: N-acetyl-cysteine; EdU: Ethynyl deoxyuridine; ROS: Reactive oxygen species; ERK1/2: Extracellularregulatedproteinkinases1/2.

## Competing interests

The authors declare that they have no competing interests.

## Authors’ contributions

XZ carried out the cell culture, performed the molecular experiments, participated in the design of the study, and drafted the manuscript. LL carried out the Immunofluorescence staining, cell proliferation and cycle assay. CC carried out Western blot analysis and the intracellular ROS measurement. Y-LC helped to draft the manuscript. X-QY participated in the design of the study. YX carried out the reagent preparation. X-TL participated in the reagent preparation and the statistical analysis. S-LG performed the statistical analysis. S-HX participated in the design of the study. M-RS helped to draft the manuscript. YS participated in the cell culture. K-MH participated in the molecular experiments. C-SZ conceived of the study, and participated in its design and coordination and helped to draft the manuscript. All authors read and approved the final manuscript.

## Authors’ information

Xi Zhang, Long Liu and Chao Chen are co-first authors.

## Supplementary Material

Additional file 1 pGC-FU-Irf-1 positive clones were identified by PCR (Identified group 1, 2, 3, 4, 5, 6, 7 were positive clones). Click here for file
