# Supplementary material for: Interferon regulatory factor-1 together with reactive oxygen species promotes the acceleration of cell cycle progression by up-regulating the cyclin E and CDK2 genes during high glucose-induced proliferation of vascular smooth muscle cells
Source: Cardiovasc Diabetol. 2013 Oct 14;12:147. doi: 10.1186/1475-2840-12-147 (PMC3852693; doi:10.1186/1475-2840-12-147)
Supplement: Additional file 1 — pGC-FU-Irf-1 positive clones were identified by PCR (Identified group 1, 2, 3, 4, 5, 6, 7 were positive clones). [file 1475-2840-12-147-S1.doc]

**pGC-FU-Irf-1 positive clones were identified by PCR (Identified group 1, 2, 3, 4, 5, 6, 7 were positive clones)**

| Reagents | Negative control group | Positive control group | Identified group 1 | Identified group2 | Identified group 3 | Identified group 4 | Identified group 5 | Identified group 6 | Identified group 7 | Identified group 8 |
| --- | --- | --- | --- | --- | --- | --- | --- | --- | --- | --- |
| Primer（＋） | 0.4µl | 0.4µl | 0.4µl | 0.4µl | 0.4µl | 0.4µl | 0.4µl | 0.4µl | 0.4µl | 0.4µl |
| Primer（—） | 0.4µl | 0.4µl | 0.4µl | 0.4µl | 0.4µl | 0.4µl | 0.4µl | 0.4µl | 0.4µl | 0.4µl |
| ddH2O | 16.2µl | 15.2µl | 15.2µl | 15.2µl | 15.2µl | 15.2µl | 15.2µl | 15.2µl | 15.2µl | 15.2µl |
| 10×buffer | 2 µl | 2 µl | 2 µl | 2 µl | 2 µl | 2 µl | 2 µl | 2 µl | 2 µl | 2 µl |
| DNTPs(2.5mM) | 0.8µl | 0.8µl | 0.8µl | 0.8µl | 0.8µl | 0.8µl | 0.8µl | 0.8µl | 0.8µl | 0.8µl |
| Taq polymerase | 0.2µl | 0.2µl | 0.2µl | 0.2µl | 0.2µl | 0.2µl | 0.2µl | 0.2µl | 0.2µl | 0.2µl |
| Colony lysate | － | 1 µl | 1 µl | 1 µl | 1 µl | 1 µl | 1 µl | 1 µl | 1 µl | 1 µl |
| Total | 20µl | 20µl | 20µl | 20µl | 20µl | 20µl | 20µl | 20µl | 20µl | 20µl |

PCR cycling conditions：

| 94℃ | 30 sec |  |  |
| --- | --- | --- | --- |
| 94℃ | 30 sec |  |  |
| 60℃ | 30 sec |  | 30 cycle  Negative Positive Marker Identified Identified Identified Identified Identified Identified Identified Identified  Control control group 1 group2 group 3 group 4 group 5 group 6 group 7 group8 |
| 72℃ | 30 sec |  |  |
| 72℃ | 6 min |  |  |


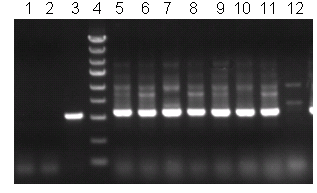


**pGCsi-FU-Irf-1 positive clones were identified by PCR**

Marker pGCsi**-Irf-1** Marker **pGCsi-Irf-1**


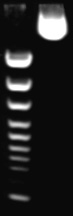

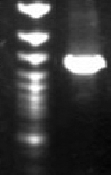


1500bp

3000bp

Plasmid extraction electrophoresis

Positive clones identified by PCR


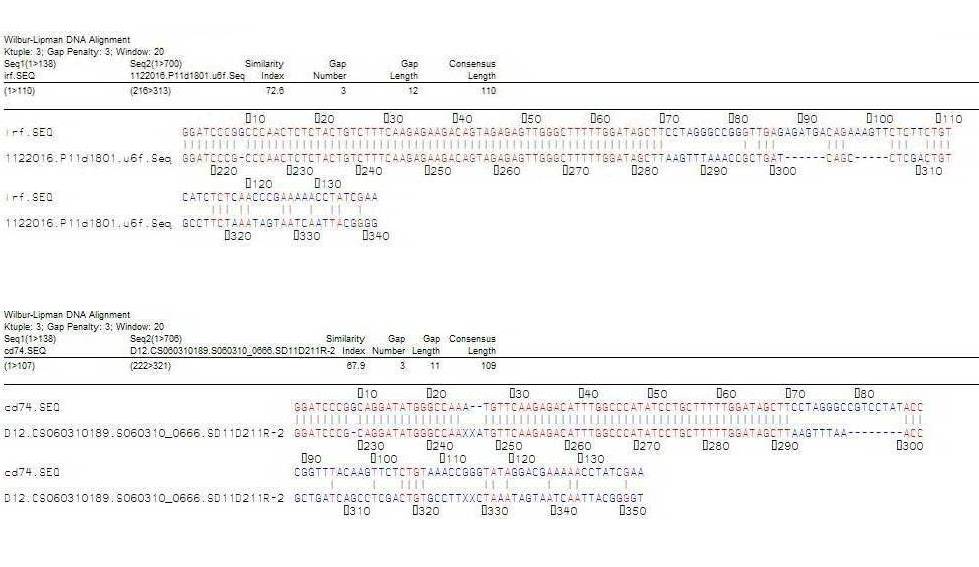


pGCsi-Irf1 sequencing comparison (100% Match)
